# Myriapoda of Canada

**DOI:** 10.3897/zookeys.819.29447

**Published:** 2019-01-24

**Authors:** David W. Langor, Jeremy R. deWaard, Bruce A. Snyder

**Affiliations:** 1 Natural Resources Canada, Canadian Forest Service, 5320–122 St. NW, Edmonton, Alberta, T6H 3S5, Canada Natural Resources Canada, Canadian Forest Service Edmonton Canada; 2 Centre for Biodiversity Genomics, University of Guelph, 50 Stone Road East, Guelph, Ontario, N1G 2W1, Canada University of Guelph Guelph Canada; 3 Department of Biological and Environmental Sciences, Georgia College and State University, 320 N. Wayne St., Milledgeville, Georgia, 31061, USA Georgia College and State University Milledgeville United States of America

**Keywords:** biodiversity assessment, Biota of Canada, centipedes, Chilopoda, Diplopoda, millipedes, Pauropoda, Symphyla

## Abstract

The currently documented fauna of described species of myriapods in Canada includes 54 Chilopoda, 66 Diplopoda, 23 Pauropoda, and two Symphyla, representing increases of 24, 23, 23, and one species, respectively, since 1979. Of the 145 myriapod species currently documented, 40 species are not native to Canada. The myriapods have not been well documented with DNA barcodes and no barcodes are available for Pauropoda. It is conservatively estimated that at least 93 additional myriapods species will be discovered in Canada: Chilopoda (40), Diplopoda (29), Pauropoda (17), and Symphyla (seven). In general, there is a serious dearth of knowledge about myriapods in Canada, and systematics research and surveys continue to be needed to help document the diversity and distribution of these groups in the country.

## Introduction

The subphylum Myriapoda contains four extant, monophyletic classes, all of which have representatives in Canada and on all continents except Antarctica: Diplopoda (millipedes), Chilopoda (centipedes), Pauropoda (pauropods), and Symphyla (garden centipedes or pseudocentipedes). Phylogenetic relationships among myriapod classes have been largely unsettled in recent years; however, the most recent phylogenomic analyses based on morphological and molecular data show strong support for Diplopoda and Pauropoda as sister groups (=Dignatha), with Symphyla most closely related to Dignatha, and Chilopoda most basal (Fernández et al. 2018).

The earliest records of myriapods from Canada are two species of millipedes described by Newport (1844) based on material in the British Museum: *Polydesmuscanadensis* (now *Pseudopolydesmuscanadensis*) and *Iuluscanadensis* (now *Uroblaniuluscanadensis*). Wood (1862) published the first record of a centipede from Canada, describing *Strigamiachionophila* based on material from Fort Simpson, Northwest Territories. This material was collected by the explorer and naturalist Robert Kennicott between 1859 and 1862 during his expedition to the Canadian north. No additional myriapod species had been recorded from Canada by 1865 as Wood (1865) listed only the three aforementioned species in his treatise on the Myriapoda of North America. Brodie and White (1883) provided the first checklist of myriapods of Canada and listed five centipede and five millipede species from the country. Three species of Chilopoda and nine of Diplopoda were collected by Geological Survey of Canada personnel (mainly by JB Tyrrell) in 1882–1883 (Chamberlin 1920). Only two of these species were included in the list of Brodie and White (1883). These collections were from British Columbia, Alberta, Ontario, and Quebec and included three millipede species new to science with two type localities in Alberta (Bow River, Waterton Lake) and one in British Columbia (Columbia Valley). Other early country records were reported by Bollman (1887) who described one species of millipede and one centipede from Glacier, British Columbia, although the dates of collection were not indicated.

In Canada, all four classes of myriapods are relatively poorly studied as there has been relatively little sampling of the fauna in the country and there hasn’t been anyone in Canada who has focused on the systematics of these groups. Diplopoda is the best known of the four classes. There are numerous Chilopoda and Diplopoda samples from Canada awaiting identification in Canadian collections. In contrast, Pauropoda and Symphyla, which are small in size and live in cryptic habitats, are very poorly represented in Canadian collections, so knowledge of the fauna and its distribution and ecology is fragmentary. All four classes of myriapods were briefly summarized in *Canada and its insect fauna* (Danks 1979), with 47 reported species of Diplopoda (Hoffman 1979), 29–31 of Chilopoda (Kevan 1979), one of Symphyla (Scheller 1979b), and none of Pauropoda (Scheller 1979a). The number of documented species in Canada has increased since 1979 for all groups. Kevan and Scudder (1989) provided illustrated keys to families of Canadian myriapods, which are still useful despite the more recent addition of several newly recorded families and a modified family structure for some Diplopoda and Chilopoda (Tables 1, 2).

All four myriapod classes are associated with soils and epigaeic habitats, and at least some Chilopoda and Diplopoda are associated with rotting wood. Centipedes are largely predaceous and are venomous (Undheim et al. 2015). The other three classes are largely detritivores, although a few millipedes are known to consume living or dead animal tissue (Hoffman and Payne 1969). Some symphylans feed on roots (fine roots and root hairs) and can damage plants, including crops (Beirne 1972).

The current synopsis is based on literature records, examination of authoritatively identified material in a few Canadian collections, and DNA barcode data in the Barcode of Life Data System (BOLD) database (Ratnasingham and Hebert 2007; http://www.boldsystems.org/). Species lists have not been included in this work but are available from the corresponding author upon request.

## Chilopoda

The statement, “The centipedes are among the least studied of the larger Canadian arthropods…” is lamentably just as true now as it was 38 years ago when Kevan (1979) wrote it to introduce his treatment of the Chilopoda of Canada. While the Canadian fauna is somewhat better known today, it is estimated that only a little more than half of the Canadian fauna is documented (Table 1). This relatively poor state of knowledge is attributed to the weak taxonomic foundation for Chilopoda in North America in general, and the paucity of effort focused on surveying and documenting the Canadian centipede fauna. These two causes are undoubtedly interlinked as the lack of a solid taxonomic foundation and identification tools for most groups of North American centipedes likely does not engender interest in the group by professional and amateur taxonomists.

**Table 1.** Census of Chilopoda in Canada.

Taxon^1^	No. species reported in Kevan (1979)^2^	No. species currently known from Canada^3^	No. BINs^4^ available for Canadian species	Est. no. undescribed or unrecorded species in Canada	General distribution by ecozone^5^	Information sources^6^
**Order Geophilomorpha**						
Geophilidae ^7^	10	16 (6)	13	10	most ecozones	Carl and Guiguet 1956; CNCI
Himantariidae	1	1 (1)	0	1	Boreal Shield	
Mecistocephalidae	0	0	0	1		
Schendylidae	1	2 (1)	5	2	Boreal Shield, Mixedwood Plain, Taiga Cordillera, Boreal Cordillera	Pereira and Hoffman 1993; CNCI
**Order Lithobiomorpha**						
Henicopidae	2	2 (1)	4	3	Pacific Maritime, Prairies, Taiga Plains, Montane Cordillera	
Lithobiidae	12	27 (3)	37	20	most ecozones	CNCI
**Order Scolopendromorpha**						
Cryptopidae	2	3 (3)	0	2	Pacific Maritime, Boreal Shield, Mixedwood Plains	Shelley 2002a
Scolopocryptopidae	1	2 (1)	0	1	Pacific Maritime, Mixedwood Plains	Shelley 2002a
**Order Scutigeromorpha**						
Scutigeridae	1	1 (1)	1	0	domiciliary in several ecozones	
**Total**	**30**	**54 (17)**	**60**	**40**		

**^1^**Classification follows that indicated in Chilobase 2.0 (Bonato et al. 2016). **^2^**This does not include species listed by Kevan (1979) as “probable”. **^3^**The numbers in parentheses represents the number of non-native species included in the total. **^4^**Barcode Index Number, as defined in Ratnasingham and Hebert (2013). BIN data were extracted from BOLD on August 20, 2018. ^5^See figure 1 in Langor (2019) for a map of ecozones. **^6^**Literature published before 1979 is usually not included, with one exception, as it was considered by Kevan (1979, 1983a). Kevan (1983a), Kevan and Scudder (1989), Behan-Pelletier (1993) and Mercurio (2010) are applicable to most families but are not listed in the table due to space considerations. CNCI refers to specimens present in the Canadian National Collection of Insects, Arachnids and Nematodes, Ottawa, Ontario. **^7^**Includes Chilenophilidae, Dignathodontidae and Linotaeniidae.

A few chilopodologists made enormous strides in the 20^th^ century to describe North American species. For example, of the 556 native species of centipedes reported from North America by Mercurio (2010), 405 were described by Ralph Chamberlin and 21 by Ralph Crabill. Unfortunately, however, there is a distinct shortage of modern taxonomic revisions, and many genera and some families remain inadequately circumscribed. One notable exception is the relatively small order Scolopendromorpha which, thanks largely to the efforts of Rowland Shelley, is relatively well studied in North America, and modern illustrated keys to species are available (e.g., Shelley 2002a). An annotated catalog of the centipedes of North America (Mercurio 2010) is of enormous help to those interested in working on this group. Furthermore, the well-illustrated key to myriapod orders and families in Canada (Kevan and Scudder 1989) is a useful resource to help those interested in chilopod identification. The on-line database, Chilobase 2.0 (Bonato et al. 2016; http://chilobase.biologia.unipd.it/) contains much current information about the classification and nomenclature of Chilopoda, but it is incomplete with respect to the distribution of the North American fauna. Thus, those interested in the general distribution of North American centipedes should consult Mercurio (2010). For the Geophilomorpha, Bonato and Minelli (2014) provide an overview of the order in Europe, which is the most current source of information about non-native species of this order in North America. Bonato et al. (2012) provide an overview of the relatively large genus *Strigamia*, which has representation in Canada, and resolves a number of taxonomic and nomenclature problems within this genus and related genera. The illustrated synopsis of anatomical terminology for centipedes is useful for those working on taxonomy and identification (Bonato et al. 2010).

In Canada, centipedes have received very little attention taxonomically or ecologically. From the publication of the first checklist of Canadian species (Brodie and White 1883), it was almost a century before the next synopsis of the Canadian fauna (Kevan 1979), although there was an earlier review of the fauna of Newfoundland (Palmén 1954), the only Canadian jurisdiction to receive such an inventory of its centipede fauna. Kevan (1983a) produced a more complete list of species known from Canada and Alaska, and this was updated by Behan-Pelletier (1993). Snyder (2014) provided a synopsis of species known and expected to be present in Canadian grasslands.

Currently, there are 54 species known to be established in Canada, including *Scutigeracoleoptrata* (Linnaeus) which is limited to human domiciles (Table 1). In comparison, there are 633 species (including non-native species) known from North America (Mercurio 2010) and 3110 species globally (Minelli 2011). The Lithobiomorpha represents the largest proportion of the Canadian fauna (53.7%), followed by Geophilomorpha (35.2%), Scolopendromorpha (9.3%) and Scutigeromorpha (1.8%), and this proportional representation is very similar to that for the North American fauna as a whole (Mercurio 2010). About 31% of the documented Canadian fauna is not native compared to 12% for the North American fauna (Mercurio 2010). Undoubtedly other non-native species are established in Canada but are yet undocumented.

Compared to the 1979 assessment, the number of species documented in Canada has increased by 24 (80%), with the greatest increases within the families Lithobiidae and Geophilidae. Most of the changes to the fauna since 1979 were reported by Kevan (1983a) and Behan-Pelletier (1993), and mainly represent Canadian species occurrences already reported in earlier literature that were missed by Kevan (1979). All of these authors, however, overlooked the records of two Geophilidae, *Cheilethakincaidi* Chamberlin and *Geophilusglyptus* (Chamberlin), recorded from Bunsby Islands, British Columbia by Carl and Guiguet (1956), the specimens of which were identified by Chamberlin. With the exception of the Scolopendromorpha, our collective knowledge of the diversity and distribution of Canadian centipedes has not increased much over the last 25 years. While most major terrestrial arthropod collections in Canada contain small-to-moderate numbers of centipede samples, the majority of those are not authoritatively identified. Most Canadian records come from southern Quebec, Ontario, British Columbia, and the island of Newfoundland, the latter thanks to the Fennoscandinavian expeditions of 1949 and 1951 as reported by Palmén (1954). All ecozones of Canada are poorly known in terms of their centipede faunas.

The number of additional species expected to be in Canada but yet undocumented (either undiscovered or undescribed) was estimated by examination of the distribution of species reported in Mercurio (2010) and references contained therein. Some species collected in the USA within 100 km of the Canadian border, and which have broad distributions in the USA (i.e., not likely to be local endemics), were deemed to be likely present in Canada and this forms the basis of the conservative estimate of undocumented species for Canada (Table 1). Thus, it is estimated that 43% of the Canadian fauna (40 species) is yet undocumented, mostly members of the families Lithobiidae and Geophilidae (Table 1).

The generation of DNA barcodes for Canadian centipedes is still in the early stages as material has been provided from only a small number of specimens and localities. Nonetheless, 60 Barcode Index Numbers (BINs; see Ratnasingham and Hebert 2013) are represented based on Canadian specimens (Table 1). In the Schendylidae, Henicopidae, and Lithobiidae there are more BINs than documented species, which may be indicative of undocumented species diversity. Clearly much work remains to fully document the Canadian fauna of centipedes.

## Diplopoda

The taxonomic foundation for reliably identifying Diplopoda found in Canada is in much better shape than for Chilopoda. Fortunately, there has been considerable taxonomic research in the USA, especially by Ralph Chamberlin, Nell Causey, Richard Hoffman, Petra Sierwald, William Shear, and Rowland Shelley, that has greatly aided knowledge of the Canadian fauna. Nonetheless, many families can benefit from modern taxonomic revisions that consider molecular and morphological characters. A catalogue of North and Middle American Diplopoda is available (Hoffman 1999) and, although now almost 20 years old and a bit dated, is still an enormously helpful resource. The on-line database Millibase (www.millibase.org/), which covers the global fauna, is also a helpful resource but is incomplete with respect to capturing published knowledge about the Canadian fauna.

As with Chilopoda, there has been a dearth of targeted survey work on millipedes in most of Canada so the fauna of all ecozones is incompletely known. The only Canadian jurisdiction that experienced a faunal inventory is the island of Newfoundland, which was extensively surveyed during the Fennoscandinavian expeditions of 1949 and 1951 (Palmén 1952). Beyond that, most current Canadian records are from southwestern British Columbia and southern Ontario and Quebec. The Canadian fauna was summarized by Hoffman (1979) who reported 47 species in 15 families and six orders; however, the species numbers were reported only at the order level and no species list was included. Furthermore, he predicted that another 22–23 species likely occurred in Canada for a total fauna of 69–70 species. Shortly thereafter, and based on literature records and authoritative examination of holdings of some Canadian collections, Kevan (1983b) published a list of 65 species known from Canada, several of which were subsequently synonymized and others identified only to genus. Shelley (1988) published a species list for eastern Canada (Ontario and eastward), including 38 species. Shelley (1990a) gave distributions for species in British Columbia. Kevan and Scudder (1989) published some faunal updates and Behan-Pelletier (1993) provided a revised list of species in Canada and their known provincial and territorial distributions. The most recent treatment of the Canadian fauna was by Shelley (2002b) who reviewed the central Canadian fauna (Alberta, Saskatchewan and Manitoba) in detail and also provided a list of the known and expected species for the entire country that included 62 recorded species and another 11 species that were considered likely in Canada based on distributions in the USA.

Currently, there are 66 described species in 18 families and six orders known in Canada (Table 2), in comparison to ~1500 species known from North America (an estimate based mainly on information in Hoffman (1999)) and 15,982 species known globally (Sierwald and Spelda 2018). The Parajulidae represents about 18.2% of the described Canadian fauna, followed by Polydesmidae (15.2%) and Julidae (12.1%). About 32% of the described Canadian fauna is non-native compared to only 2% for the North American fauna (Snyder and Hendrix 2008); however, this high proportion is likely because a large portion of the native Canadian fauna is unknown. The current species total is a 40% increase over that reported by Hoffman (1979), highlighting a substantial increase in knowledge of the fauna over the last ca. 40 years. In addition to described species, material has been collected in Canada representing four additional families (Glomeridesmidae, Striariidae, Tingupidae, and Urochordeumatidae; Table 2) but it is not known if this material represents described species. Taking into account this unidentified material, the opinion of Shelley (2002b) concerning species likely to be present in Canada, and supplemented by examination of species distributions documented in subsequent publications about the North American fauna, we conservatively estimate that at least 29 additional species reside in Canada, including seven additional families. This means that Canada should have a millipede fauna of at least 95 species and is thus roughly equivalent to the estimated species richness of the Canadian centipede fauna (94 species; Table 1). However, as there are about three times as many millipedes as centipedes known from North America (Snyder and Hendrix 2008, Mercurio 2010), the Canadian millipede fauna is likely to be much more diverse than estimated herein.

**Table 2.** Census of Diplopoda in Canada.

Taxon^1^	No. species reported in Hoffman (1979)^2^	No. species currently known from Canada^3^	No. BINs^4^ available for Canadian species	Est. no. undescribed or unrecorded species in Canada	General distribution by ecozone^5^	Information sources^6^
**Order Polyxenida**						
Polyxenidae	1	2 (1)	2	0	Atlantic Maritime, Mixedwood Plains, Pacific Maritime	
**Order Glomeridesmida**						
Glomeridesmidae	0	0	0	1	Pacific Maritime	Shelley et al. 2007a
**Order Polyzoniida**						
Hirudisomatidae	?	1	2	0	Pacific Maritime	Shelley 1995, 1996
Polyzoniidae	?	2	1	0	Boreal Shield, Mixedwood Plains	
**Sub-total Polyzoniida**	**2**	**3**	**3**	0		
**Order Julida**						
**Superfamily Blaniuloidea**						
Blaniulidae	?	5 (5)	2	0	widespread south of taiga ecozones	Shelley and Smith 2011
Okeanobatidae	?	1	0	0	Boreal shield, Mixedwood Plains	
**Superfamily Juloidea**						
Julidae	?	8 (8)	26	1	widespread south of taiga ecozones	Shelley and Whitney 1994, Shelley and Smith 2011
**Superfamily Nemasomatoidea**						
Nemasomatidae	?	1	3	1	Montane Cordillera, Pacific Maritime	Enghoff 1985
**Superfamily Paeromopodoidea**						
Paeromopodidae	0	0	0	2	Montane Cordillera, Pacific Maritime	
**Superfamily Parajuloidea**						
Parajulidae	?	12	1	4	widespread south of taiga ecozones	Carl and Guiguet 1956, Shelley and Smith 2016
**Total Julida**	**20**	**27 (13)**	**32**	**8**		
**Order Spirobolida**						
Spirobolidae	1	1	1	0	Boreal Shield, Mixedwood Plains	Shelley et al. 2006
**Order Spirostreptida**						
Cambalidae	0	0	0	2	Pacific Maritime	
**Order Callipodida**						
Abacionidae	0	0	0	2	Mixedwood Plains	
**Order Chordeumatida**						
**Suborder Craspedosomatidea**						
**Superfamily Anthroleucosomatoidea**						
Anthroleucosomatidae	0	0	0	1	Pacific Maritime	
**Superfamily Brannerioidea**						
Branneriidae	0	0	0	1	Mixedwood Plains	
Microlympiidae	0	0	0	1	Pacific Maritime	
Tingupidae	0	0	0	1	Pacific Maritime	Shear and Shelley 2007, Shelley et al. 2009b
**Superfamily Cleidogonoidea**						
Cleidogonidae	0	0	4	2	Mixedwood Plains	
Trichopetalidae	?	1	0	0	Atlantic Maritime, Mixedwood Plains, Newfoundland Boreal	Shear 2010
**Superfamily Craspedosomatoidea**						
Craspedosomatidae	?	1 (1)	2	0	Mixedwood Plains	Shelley 1990b, Hoffman 1999
**Suborder Heterochordeumatidea**						
**Superfamily Heterochordeumatidea**						
Conotylidae	?	6	0	3	widespread south of taiga ecozones	Shelley and LeSage 1996, Shear 2004, Shelley et al. 2009a
**Suborder Striadiidea**						
**Superfamily Caseyoidea**						
Caseyidae	?	4	0	0	widespread south of taiga ecozones	Shelley 1993, Shelley et al. 2007b, 2009
Urochordeumatidae	0	0	0	1	Pacific Maritime	CNCI
**Superfamily Stiarioidea**						
Rhiscosomididae	?	1	0	0	Pacific Maritime	Shelley 1990a, Shelley et al. 2009a
Striariidae	0	0	0	1	Pacific Maritime	
unplaced Chordeumatida			7			
**Total Chordeumatida**	**6**	**17 (1)**	**13**	**6**		BOLD^7^
**Order Polydesmida**						
**Suborder Leptodesmidea**						
**Superfamily Xystodesmoidea**						
Xystodesmidae	?	6	6	3	Mixedwood Plains, Montane Cordillera, Pacific Maritime,	Shelley 1990a, Shelley et al. 2009a, Marek et al. 2014, Shelley and Smith 2018
**Suborder Polydesmidea**						
**Infraorder Polydesmoides**						
**Superfamily Polydesmoidea**						
Macrosternodesmidae	0	3 (1)	2	0	Pacific Maritime, Montane Cordillera, Newfoundland Boreal	Carl and Guiguet 1956, Whitney and Shelley 1995, Shelley 1994
Polydesmidae	?	10 (4)	4	2	widespread south of taiga ecozones	Judd 1967, Shelley 1996, 2007, Shelley and Smith 2011, Shelley and Snyder 2012
**Suborder Strongylosomatidea**						
Paradoxosomatidae	0	1 (1)	2	0	Atlantic Maritime, Mixedwood Plains, Newfoundland Boreal, Pacific Maritime	Judd 1967, Shelley 1990a
**Total Polydesmida**	**17**	**20 (6)**	**14**	**5**		
**Total**	**47**	**66 (21)**	**65**	**29**		

**^1^**Classification follows that indicated in Millibase (www.millibase.org/index.php) with the exception that Nearctodesmidae is included as a subfamily of Macrosternodesmidae which is placed in Polydesmoidea as suggested by Shear and Reddell (2017). **^2^**Hoffman (1979) provided species tallies only by order. Thus, many family level tallies are not available. **^3^**The numbers in parentheses represents the number of non-native species included in the total. **^4^**Barcode Index Number, as defined in Ratnasingham and Hebert (2013). BIN data were extracted from BOLD on August 20, 2018. **^5^**See figure 1 in Langor (2019) for a map of ecozones. **^6^**Literature published before 1979 is usually not included, with one exception, as it was considered by Hoffman (1979) and Kevan (1983b). Kevan (1983b), Kevan and Scudder (1989), Behan-Pelletier (1993), Hoffman (1999), and Shelley (1988, 2002b) are applicable to most families but are not listed in the table due to space considerations. CNCI refers to specimens present in the Canadian National Collection of Insects, Arachnids and Nematodes, Ottawa, Ontario. **^7^**BOLD: Barcode of Life Data Systems (http://www.boldsystems.org/).

Only 65 BINs representing 14 of the 22 families of millipedes known from Canada are available (Table 2), and only 12 BINs are associated with material identified to species level. Clearly, much work remains to adequately barcode Canadian millipedes. Notably, 26 BINs are associated with Julidae, a family not native to Canada, which is much higher than the eight documented species recorded from Canada.

The ecology of millipedes has received little attention in Canada; however, in a study of the influence of *Harpaphehaydeniana* Wood on litter decomposition in the coastal forests of British Columbia, Cárcamo et al. (2000) found that this species consumed as much as 36% of the annual litter fall.

## Pauropoda

Pauropods are soft-bodied, small (0.5–2.0 mm long) detritivores found in soils (Scheller 1979a). Worldwide there are about 835 known species (Scheller 2011a), and about 100 species are known from the USA (Scheller 2011b), however, the fauna is poorly documented at both regional and global scales (Scheller 2011a, b). The Canadian fauna is poorly known in terms of species composition, distribution, and ecology, although some progress has been made since 1979. The earliest record from Canada is from the Yukon where Hilton (1931) described a new species, *Stylopauropusdawsoni*; however, as his type material is considered lost and the description is very superficial, this species is considered to be *nomen dubium* (Scheller 1984). Scheller (1979a) reported no named species from Canada but estimated that around 20 species could be found there. Based on examination of 320 specimens from three Canadian collections in British Columbia, Ontario and Quebec, Scheller (1984) reported 23 species in two families (Table 3) from those three provinces, including six species new to science, and also provided keys to known families and genera in Canada. It is possible that four species are non-native based on known distribution. Six additional species have wide distributions (cosmopolitan in some cases) and some of these may also be non-native in Canada. This material could not be located in Canadian collections so may still be in the private collection of Ulf Scheller in Sweden. No new species have since been reported from the country. Also, no Canadian specimens of Pauropoda have been DNA barcoded.

**Table 3.** Census of Pauropoda in Canada.

Taxon^1^	No. species currently known from Canada^2^	No. BINs^3^ available for Canadian species	Est. no. undescribed or unrecorded species in Canada	General distribution by ecozone^4^	Information sources
**Order Tetromerocerata**					
Brachypauropodidae	1 (0)	0	4	Pacific Maritime	Scheller 1984, 1985, 1986a
Eurypauropodidae	0	0	2		Scheller 1985
Pauropodidae	22 (4)	0	11	Boreal Shield, Mixedwood Plains, Pacific Maritime, Western Interior Basin	Scheller 1984, 1985, 1986a
**Total**	**23 (4)**	**0**	**17**		

**^1^**Classification that of Scheller (2008). **^2^**The numbers in parentheses represents the number of non-native species included in the total. **^3^**Barcode Index Number, as defined in Ratnasingham and Hebert (2013). BIN data were extracted from BOLD on August 20, 2018. **^4^**See figure 1 in Langor (2019) for a map of ecozones.

Based on a survey of the literature treating Pauropoda in the continental USA (Scheller 1985 and references therein) and Alaska (Scheller 1986a), it is conservatively estimated that at least 17 additional species and one additional family (Eurypauropodidae) will be found in Canada (Table 3).

It is clear from a quick inventory of some major Canadian collections that pauropods have been seldom collected and preserved in Canada as there is little material accessioned. Records exist for only British Columbia, Ontario, Quebec and Yukon. In Alberta, the Alberta Biodiversity Monitoring Institute has been conducting a systematic survey of soil fauna across the entire province on a 20 km × 20 km grid since 2007. In support of this provincial-scale survey, taxonomists at the Royal Alberta Museum extract approximately 800 soil samples each year for invertebrates, particularly oribatid mites. A recent census of residual material from 194 of these samples yielded no Pauropoda or Symphyla (T Cobb pers. comm.), underscoring the difficulty in collecting these organisms using soil cores. By comparison, Chilopoda and Diplopoda were extracted from about 2% of samples.

## Symphyla

Symphyla are also small (1–10 mm long) soil-dwellers and are usually infrequently collected. However, the most wide-spread species in Canada, the non-native and cosmopolitan *Scutigerellaimmaculata* (Newport), can be abundant in greenhouses and outdoors in more moderate climates and can cause significant damage to roots of many vegetable crops especially in southern British Columbia and Ontario (Beirne 1972).

Symphyla is the least diverse class of myriapods with about 35 species known from North America (Scheller 1986b) and 195 globally (Szucsich and Scheller 2011). Scheller (1979b) reported one species from Canada, *S.immaculata*, which is now likely to be distributed across southern Canada from coast to coast (Beirne 1972, Morris and Morry 1983). Subsequently, Kevan (1983a) reported the cosmopolitan and likely introduced *Symphylellavulgaris* (Hansen) based on a specimen in the Lyman Entomological Museum (McGill University) collected from a southern Quebec hardwood forest. Since then, no more species have been recorded from Canada, although undoubtedly additional species occur here. In addition to reporting one species, Scheller (1979b)estimated about 10 undocumented species in Canada. Kevan (1983b) mentions seven species and one more family (Geophilellidae) that are likely in Canada (Table 4) and we adopt his estimate herein. Only four specimens from Canada have been DNA barcoded and each represents a different BIN, three within Scolopendrellidae and one within Scutigerellidae (Table 4).

**Table 4.** Census of Symphyla in Canada.

Taxon^1^	No. species reported in Scheller (1979b)	No. species currently known from Canada^3^	No. BINs^2^ available for Canadian species	Est. no. undescribed or unrecorded species in Canada	General distribution by ecozone^3^	Information sources^4^
**Order Cephalostigmata**						
Geophilellidae	0	0	0	2		Kevan 1983a
Scolopendrellidae	0	1 (1)	3	1	Mixedwood Plains	Kevan 1983a
Scutigerellidae	1	1 (1)	1	4	likely all ecozones south of taiga and Boreal Cordillera	Beirne 1972, Kevan 1983a, Morris and Morry 1983; BOLD
**Total**	**1**	**2 (2)**	**4**	**7**		

**^1^**Classification follows that of Millibase (www.millibase.org/index.php). **^2^**Barcode Index Number, as defined in Ratnasingham and Hebert (2013). BIN data were extracted from BOLD on August 20, 2018. **^3^**See figure 1 in Langor (2019) for a map of ecozones. **^4^**BOLD: Barcode of Life Data Systems (http://www.boldsystems.org/).

## Gaps and opportunities

Given the paucity of knowledge about the faunal composition, taxonomy, distribution, and ecology of all myriapod classes in Canada, there are plentiful opportunities to add to this body of knowledge by collecting and studying these fascinating creatures. All myriapod classes are poorly sampled over all of Canada, meaning that any specimens encountered are likely to represent useful records. Even the North may have considerable diversity, especially in Beringian areas. Centipedes and millipedes are frequently encountered by turning rocks and logs, picking apart highly rotten logs, sifting dead leaves, and using pitfall traps. Pauropoda and Symphyla are much less frequently encountered or detected. Sometimes rolling deeply embedded rocks will reveal specimens of these two classes, and sifting of litter is a useful approach. Tullgren and Berlese funnel extractions of organic and mineral soil layers may also yield specimens. We implore those who encounter myriapods to make an effort to preserve specimens in ethanol and accession them into a publically accessible collection. The other challenge with working with myriapods is the poor state of taxonomy and relative paucity of taxonomic resources and local expertise. Diplopoda has a much better taxonomic foundation and better availability of taxonomic resources than the other groups. In North America there are a few people who actively study taxonomy of millipedes. For centipedes there is very little taxonomic work ongoing in North America and for Pauropoda and Symphyla there is essentially none. We encourage others to seek out, observe, collect and study these fascinating creatures in Canada and more broadly in North America.
